# Dural-Based Cavernoma of the Posterior Cranial Fossa Mimicking a Meningioma: A Case Report

**DOI:** 10.7759/cureus.560

**Published:** 2016-04-06

**Authors:** Aurora S Cruz, Shiveindra Jeyamohan, Marc Moisi, R. Shane Tubbs, Jeni Page, Parthasarathi Chamiraju, Lara Tkachenko, Steven Rostad, David W. Newell

**Affiliations:** 1 Neurological Surgery, University of Louisville; 2 Neurological Surgery, University of California, Irvine; 3 Neurosurgery, Swedish Neuroscience Institute; 4 Neurosurgery, Seattle Science Foundation; 5 Neurosurgery, Wayne State University School of Medicine; 6 Pathology, CellNetix

**Keywords:** cavernoma, meningioma, posterior fossa lesion, cavernous malformation

## Abstract

Cavernous angiomas usually occur in the parenchyma of both the supra and infratentorial compartments. At times, they can both clinically and radiologically mimic other dural-based lesions. We present a case of a patient with chronic occipital headaches, initially thought to have a meningioma, but proven to be a cavernoma with histological analysis.

## Introduction

Cavernous angiomas, also known as cavernous malformations or cavernomas, are benign vascular lesions usually found in the brain parenchyma. With no gender predisposition, they affect between 0.4% to 0.6% of the population [1]. Parenchymal cavernomas present differently both clinically and radiologically, and can mimic other dural-based lesions such as meningioma. This ambiguity may lead to unexpected gross or histologic results in the operating room. Our report details the case of a patient with a suspected meningioma who underwent resection and was unexpectedly found to have a dural-based cavernous angioma. Informed consent was obtained from the patient for this study.

## Case presentation

**Figure 1:**
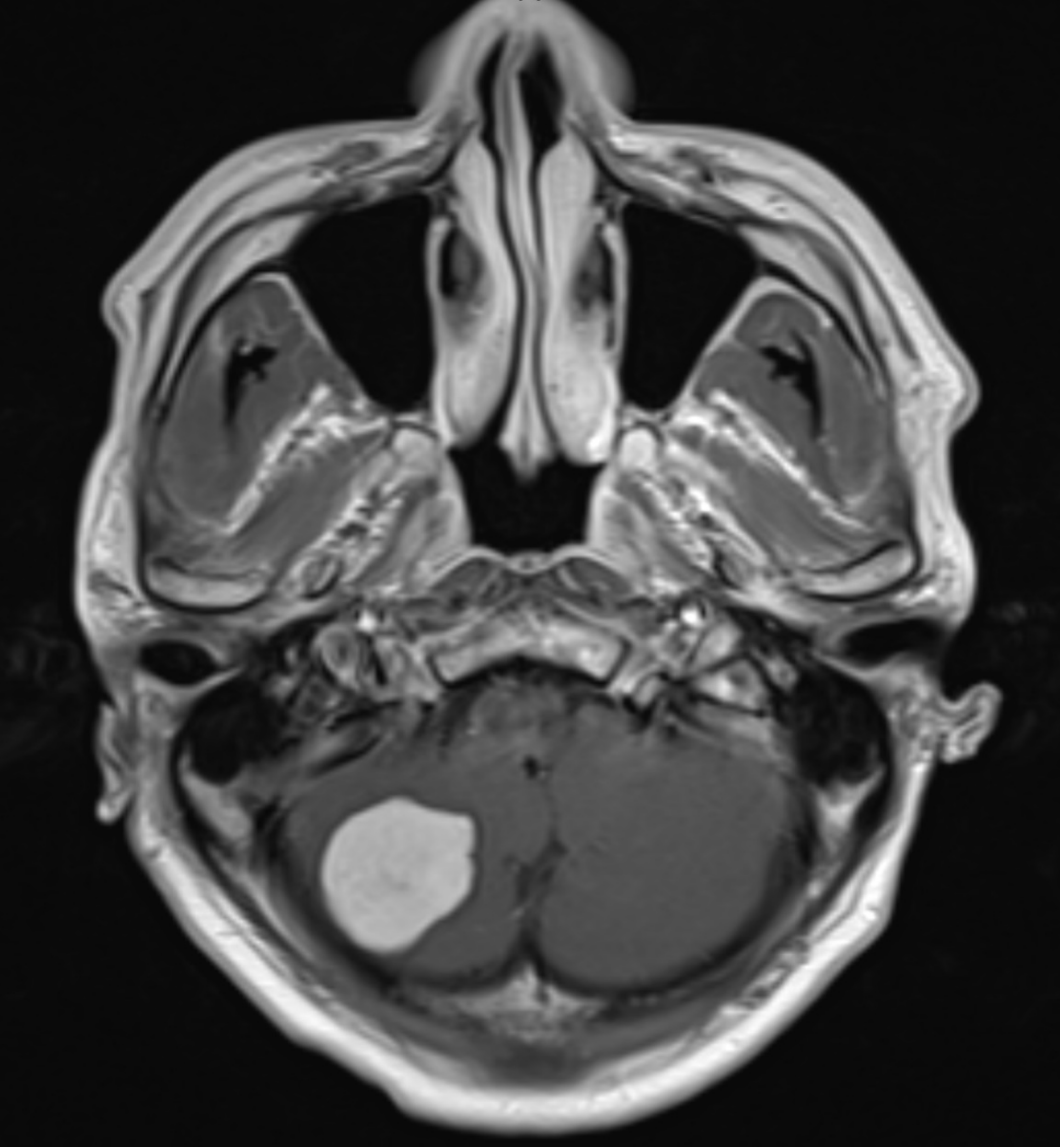
Axial View - MRI Enhanced T1-weighted axial view illustrating the lesion in the right cerebellum.

**Figure 2:**
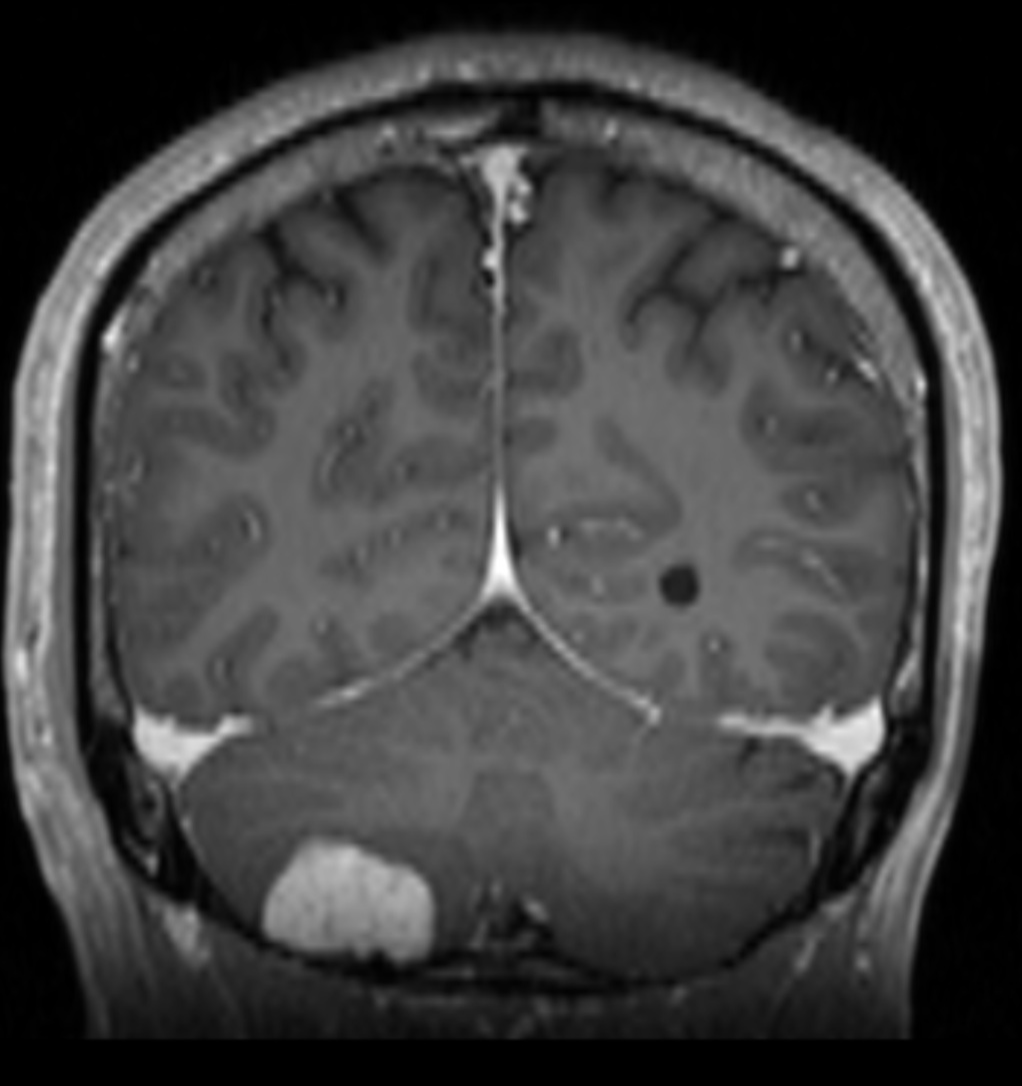
Coronal View - MRI Enhanced T1-weighted coronal view illustrating the lesion.

**Figure 3:**
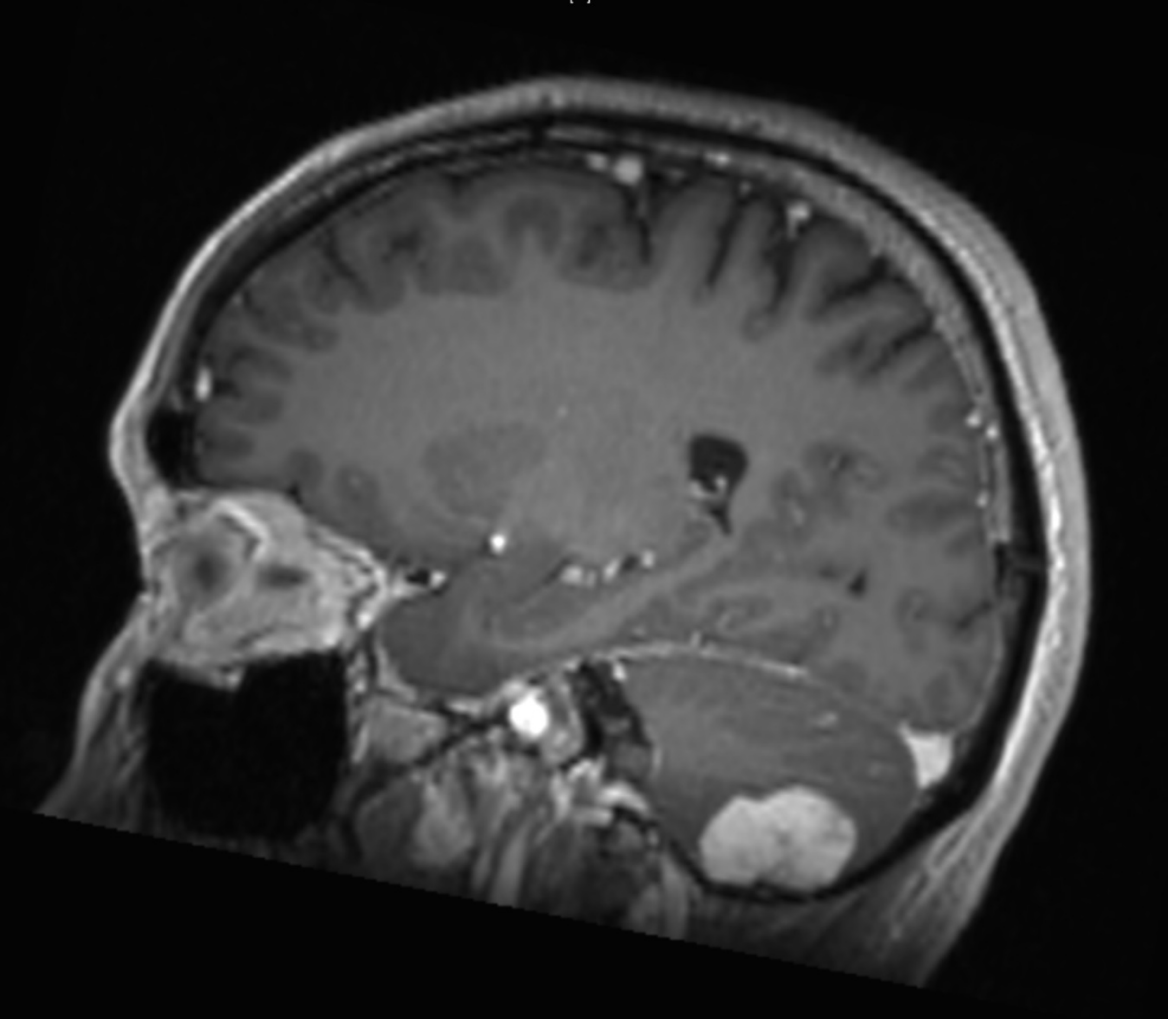
Sagittal View - MRI Enhanced T1-weighted sagittal view illustrating the lesion.

A 42-year-old man presented with longstanding occipital headaches since childhood, which progressively worsened over the preceding six months. He denied diplopia, ataxia, nausea, or weakness in his arms or legs. On physical exam he was neurologically intact. Evaluation included computed tomography (CT) scan as well as magnetic resonance imaging (MRI) of the brain. The MRI revealed a 2.8 cm, enhancing, extra-axial mass along the floor of the right posterior cranial fossa that is demonstrated in Figures 1-3.

It was recommended to the patient that he undergo elective craniotomy and resection. 

Following a right suboccipital craniotomy, the lesion was noted to be encapsulated and crimson in color. There were no significant adhesions or evidence of invasion of the brain parenchyma and the lesion was easily removed en bloc and without complications. 

**Figure 4:**
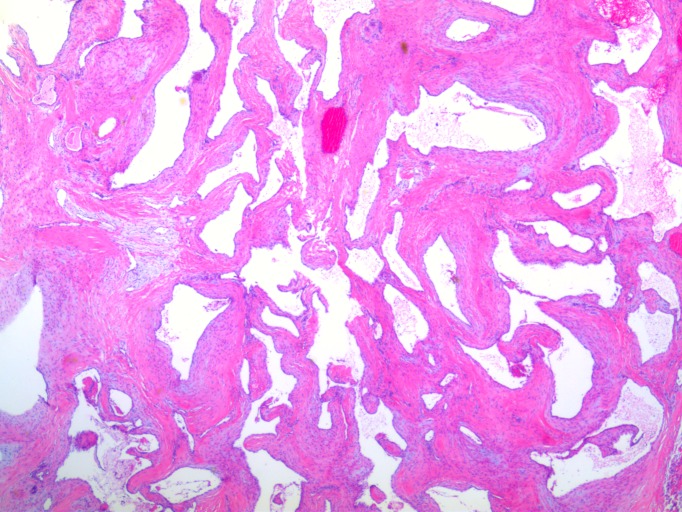
Hematoxylin and Eosin Stain Slide demonstrating multiple back-to-back vascular channels. Each vessel is lined by endothelial cell layer and smooth muscle.

**Figure 5:**
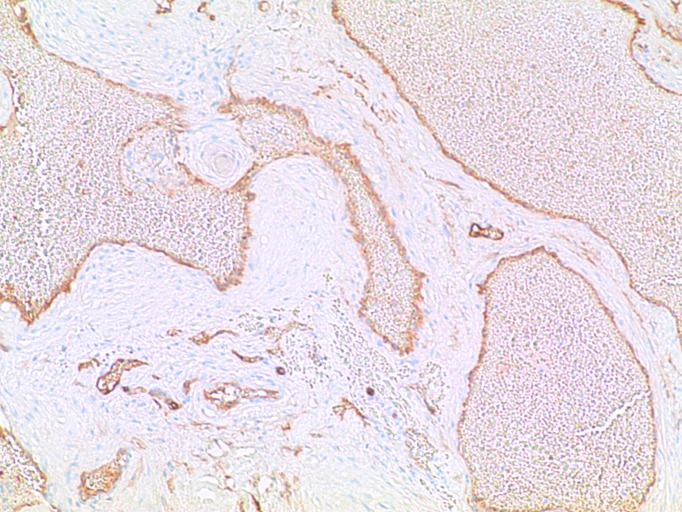
CD 31 Immunostain CD 31 immunostain decorates the edothelial lining of vascular channels.

Histological analysis of the tumor demonstrated back-to-back, thick-walled, endothelial-lined, venous channels consistent with a dural-based cavernous angioma shown in the hematoxylin and eosin stain in Figure 4. Thick walls composed of markedly hyperplastic smooth muscle cells consistent with arterialization were also found and shown in Figure 5. Focal whorl-like structures were seen; however, these lacked somatostatin receptor 2A and progesterone receptor staining typical of meningiomas and were instead positive for smooth muscle actin.

Postoperatively, the patient did very well and was discharged home on postoperative day two. At three months follow-up, he continues to do well and has complete resolution of his preoperative symptoms.

## Discussion

Cavernous angiomas are benign vascular lesions comprised of enlarged, clustered, sinusoidal vessels without intervening parenchymal tissue and that are lined with epithelium [2]. Cavernous angiomas are most commonly found in the brain parenchyma but they may also be found in the spinal cord or in an extra-axial location [2-3]. The most common presenting symptoms are seizures (37%), hemorrhage (36%), headaches (23%), and focal neurological deficits (22%) [3]. The hemorrhage rate for cavernous angiomas varies among different authors but a recent review of the literature found that the annual risk of hemorrhage was between 2.4 to 3.1% per year, with a significantly higher risk of repeat bleeding for previously hemorrhaged lesions [3]. Histologically, cavernous angiomas appear as a cluster of irregular, hyalinized sinusoidal and vascular spaces resembling a mulberry [2]. Lack of neural tissue between these vascular spaces is a defining characteristic and histologically differentiates them from telangiectasias [2, 4]. Macrophages and hemosiderin staining from asymptomatic micro-hemorrhages are also a common finding. Full excision of surrounding epileptogenic hemosiderin staining has been found to decrease postoperatively [5-6].

Cavernous angiomas have a very distinctive appearance on MRI and on CT, often have punctate calcifications [7]. The characteristic “popcorn” lesion on both T1 and T2-weighted MRI appears as lobulated, vascular spaces with a mixed signal density representing breakdown products of numerous microhemorrhages [8-9]. A peripheral ring of hypointensity seen on MRI represents hemosiderin deposited in the surrounding parenchyma [4, 7, 10]. Susceptibility Weighted Imaging (SWI) MRI sequences are more sensitive and reliable than regular MRI sequences in detecting microhemorrhages and calcifications [7].

A few cases of dural-based cavernous malformations mimicking meningiomas or other neoplastic processes have been reported in the literature [11-13]. The differential diagnosis is wide, and can include hemangiopericytomas, hemangioblastomas, solitary fibrous tumors, dural-based metastases, and angiomatous meningiomas. Dural-based meningiomas have been reported in the cavernous sinus and middle cranial fossa, and tend to be more vascular with a more aggressive clinical course when compared to convexity or infratentorial cavernomas [14]. Unlike parenchymal lesions, dural-based cavernous angiomas most commonly present with headaches, rather than seizures or hemorrhage [14]. Dural-based cavernous angiomas are histologically nearly identical to their intraparenchymal counterparts although they tend to lack calcification [15]. Radiologic interpretation, however, proves very different from intraparenchymal cavernomas. As noted in the literature, these lesions tend to be iso- to hypointense on T1-weighted images, mixed to hyperintense on T2-weighted images, contrast enhancing, and hyperdense on CT [4, 10]. These characteristics are also typical for meningiomas, thereby clouding the differential diagnosis based on history and radiologic analysis. This distinction is further clouded by the presence of dural tails, hyperostotic reactions, and perilesional edema noted with some dural-based cavernous angiomas [2, 11, 13, 16]. Grossly, the lesion in our case was well encapsulated with the fibrous consistency of a meningioma. Until proven by pathology, we were convinced, based on imaging and intraoperative visualization, that the tumor was a meningioma.

Previous cases have described lesions similar to ours with cavernous angiomas being mistaken for neoplastic lesions such as meningiomas and also neoplastic lesions mistaken preoperatively for cavernous angiomas [11-13, 16]. This lack of radiologic and clinical distinction can lead to unexpected findings in the operating room and may alter treatment plans, especially in the case of misidentified meningiomas. Given the lack of differentiable factors, it is important intraoperatively for the neurosurgeon to keep in mind the possibility of dural-based cavernous angiomas when formulating a differential diagnosis and choosing the appropriate excision technique and postoperative treatment plan. These dural-based cavernomas tend not to recur, and can safely be monitored without adjunct therapy [1, 3].

## Conclusions

With the advancement of radiological techniques, the preoperative diagnosis of intracranial lesions continues to improve. However, as in the case reported herein, the definitive diagnosis continues to be the pathological specimen.
